# Recent Advances in the Pursuit of an Effective *Acinetobacter baumannii* Vaccine

**DOI:** 10.3390/pathogens9121066

**Published:** 2020-12-19

**Authors:** Patrick S. Gellings, Ashley A. Wilkins, Lisa A. Morici

**Affiliations:** 1 Department of Microbiology and Immunology, Tulane University School of Medicine, New Orleans, LA 70112, USA; pgellings@tulane.edu (P.S.G.); awilk3@lsuhsc.edu (A.A.W.); 2 Department of Microbiology and Immunology, Louisiana State University Health Sciences Center, Shreveport, LA 71103, USA

**Keywords:** *Acinetobacter baumannii*, antimicrobial resistance, SARS-CoV-2, outer membrane vesicles, live-attenuated vaccine, subunit vaccine

## Abstract

*Acinetobacter baumannii* has been a major cause of nosocomial infections for decades. The absence of an available vaccine coupled with emerging multidrug resistance has prevented the medical community from effectively controlling this human pathogen. Furthermore, the ongoing pandemic caused by SARS-CoV-2 has increased the risk of hospitalized patients developing ventilator-associated pneumonia caused by bacterial opportunists including *A. baumannii*. The shortage of antibiotics in the development pipeline prompted the World Health Organization to designate *A. baumannii* a top priority for the development of new medical countermeasures, such as a vaccine. There are a number of important considerations associated with the development of an *A. baumannii* vaccine, including strain characteristics, diverse disease manifestations, and target population. In the past decade, research efforts have revealed a number of promising new immunization strategies that could culminate in a safe and protective vaccine against *A. baumannii*. In this review, we highlight the recent progress in the development of *A. baumannii* vaccines, discuss potential challenges, and propose future directions to achieve an effective intervention against this human pathogen.

## 1. Introduction to *A. baumannii*

*Acinetobacter* are non-motile Gram-negative coccobacilli that are found ubiquitously in nature, but are predominantly found in soil, water, and sewage [1]. There are over 50 different species of *Acinetobacter*, most of which are non-pathogenic [2]. Of the few pathogenic species, a cluster of phylogenetically related species, known as the *A. calcoaceticus-baumannii* (ACB) complex, cause the majority of infections [3]. Because *A. baumannii* is considered the most prominent and virulent, infections caused by the ACB complex are often simply referred to as *A. baumannii* infections [3]. *A. baumannii* is predominately a nosocomial, opportunistic pathogen that has the potential to cause severe disease and death. For decades, the medical community has observed *A. baumannii* develop new mechanisms to resist antimicrobial treatments, leading to fewer clinically-effective therapeutics. Because of its extensive multidrug resistance, development of prophylactic vaccines to protect at-risk populations has received considerable investigation with several promising vaccine platforms reported in pre-clinical studies during the last decade. In this review, we will briefly summarize the disease manifestations, transmission, and antimicrobial resistance mechanisms of *A. baumannii*, followed by an in-depth review of the aforementioned vaccine studies.

### 1.1. Disease Manifestations

*A. baumannii* is primarily considered an opportunistic pathogen that most commonly affects hospitalized patients, immunocompromised individuals, and military personnel with battlefield wounds [4,5,6,7,8,9], especially in humid climates [10]. *A. baumannii* infections are associated with organs and organ systems that have a high fluid exchange rate such as the urinary tract, respiratory tract, and peritoneal system [11]. The bacteria will most commonly cause pneumonia, sepsis, skin and soft tissue infections, and meningitis [11]. It has been estimated that *A. baumannii* is responsible for nearly 12% of all hospital-acquired infections worldwide, but rates for individual countries can be as low as 1–2% in some northern European countries and as high as one third of all cases in Asian countries [12,13,14,15].

Hospital-acquired pneumonia is one of the most common clinical manifestations of *A. baumannii* infections and occur most commonly in patients receiving mechanical ventilation in an intensive care setting [6,15,16,17,18]. These pneumonia cases can be difficult to treat, especially because of the prevalence of antibiotic resistance. A recent meta-analysis of 126 studies encompassing 29 countries determined that multidrug-resistant *A. baumannii* was present in 80% of all hospital-acquired and ventilator-associated pneumonia cases worldwide [19]. Serious Covid-19 cases result in hospitalizations and patients requiring mechanical ventilation. A number of retrospective studies have identified a noteworthy percent of Covid-19 patients suffering from co-infections either before or after developing Covid-19 symptoms and hospital admission. The most common culturable bacterial pathogens in Covid-19 patients appear to be *Klebsiella pneumonia* [20,21,22], *Legionella pneumophilia* [21,22,23], *Enterobacter cloacae* [24], *Staphylococcus aureus* [22,25], *Streptococcus pneumoniae* [25], and *A. baumannii* [20,21,22,24,25,26,27,28].

Sepsis is another serious complication associated with *A. baumannii* infections [29,30,31]. For every hour treatment is delayed, septic patients infected with *A. baumannii* have a 5–10% increase in mortality [31]. Sepsis cases caused by *Acinetobacter* species accounted for the third highest crude mortality rates in ICUs, with mortality rates exceeding 50% in some studies and were only surpassed by *Pseudomonas aeruginosa* and *Candida* species infections [32]. A retrospective cohort study determined that approximately 1% of all sepsis cases analyzed from a hospital had a blood culture that was positive for *A. baumannii* [29]. Of those cases, 82.3% of cultures were considered multi-drug resistant and 16.8% were extensively drug resistant [29,30].

Bacteria are the main cause of the 14 million outpatient and 850,000 inpatient skin and soft-tissue infections (SSTIs) each year [33]. Members of the ESKAPE (*Enterococcus faecium*, *Staphylococcus aureus*, *Klebsiella pneumoniae*, *Acinetobacter baumannii*, *Pseudomonas aeruginosa*, and *Enterobacter* species) family make up the bulk of SSTIs, with *A. baumannii* a leading cause of SSTIs that occur within a military environment [4,31,34]. Over the last 10–15 years, the U.S. military has recorded a significant increase in MDR *A. baumannii* infections with up to 83% of SSTIs caused by *A. baumannii* associated with combat zone operations [4,31]. Multidrug-resistant (MDR) *A. baumannii* infections are present in both trauma and burn victims as these injuries often result in secondary infections [31].

Finally, meningitis is another serious complication of *A. baumannii* infections. Nosocomial cases of *A. baumannii* meningitis are often caused by head trauma, leaking cerebrospinal fluid, or external ventricular drainage [35,36,37]. Mortality rates of nosocomial meningitis caused by *Acinetobacter* species can reach 40% in developed countries and up to 70% in developing countries [38].

### 1.2. Transmission

*A. baumannii* can be spread via aerosol droplets, person-to-person contact through sloughing of skin, sputum, urine, or feces, or through contact with a fomite [39,40,41,42,43]. Fomite spread is particularly problematic in a hospital setting. A study that observed over 250 patient interactions discovered culturable *A. baumannii* on the hands or gloves of healthcare workers in 30.3% of interactions [44]. Further, both antibiotic-resistant and antibiotic-susceptible *A. baumannii* strains are easily transferred from examination gloves made of nitrile to polypropylene plastic surfaces where the bacteria can be transferred to another healthcare worker or to a patient [45]. A separate study found carbapenem-resistant *Acinetobacter baumannii* (CRAB) present in the sputum of ventilator patients suggesting that, despite the closed nature of a ventilation system, the bacteria could still be readily spread in droplets emitted by CRAB patients [40].

The ease of *A. baumannii* spread is largely due to three separate traits: resistance to desiccation, the ability to form biofilms on abiotic surfaces, and aptitude for adhering to host cells. In fact, *A. baumannii* has been found to be viable after 2 months on an abiotic surface in a controlled laboratory setting, but endemic strains are believed to be able to survive for up to three years in inhospitable environments [46,47]. The ability of *A. baumannii* to survive for long periods of time without hydration is largely attributed to its outer polysaccharide capsule [48,49,50,51] and its ability to form biofilms to resist desiccation [52,53,54,55]. The thick polysaccharide capsule enables the bacteria to develop biofilms which retain moisture and persist for long periods of time [51]. The *A. baumannii* capsule is produced from a rich diversity of capsule loci, with over 100 distinct capsule types identified to date. The capsules can contain unique glycans with atypical acetylation sites or may be exclusively decorated with aminosugars [56,57]. The presence of RecA, a main player in homologous recombination and SOS mutagenesis, also contributes to the ability of *A. baumannii* to survive desiccation [58]. Bacterial DNA is routinely damaged during desiccation and RecA is known to repair DNA during rehydration and to confer selective advantages to the bacteria [58]. It was previously shown that a single round of desiccation and rehydration, or heat-shock of *A. baumannii* resulted in a 50-fold increase in the number of genomic mutations which was dependent on the presence of RecA [58].

### 1.3. Antimicrobial Resistance

Antimicrobial resistance (AMR) among bacteria has become a global crisis due to innate or acquired resistance mechanisms and has caused an estimated two million illnesses and 23,000 deaths to date [59]. By 2050, at least 10 million individuals are expected to die from multidrug-resistant organisms unless the medical and scientific communities make a substantial investment in research focused on understanding antibiotic resistance and developing new or alternative therapies [59]. While AMR is quickly becoming an issue for many bacteria, it is especially dire for *A. baumannii.* The CDC recently reclassified CRAB from its previous status of Hazard Level Serious to Hazard Level Urgent, meaning that the level of antibiotic resistance requires aggressive action and poses a notable threat to public health [59]. According to the CDC, CRAB alone has resulted in $281 million dollars in healthcare costs and contributed to an estimated 8,500 hospitalizations and 700 deaths in the US in 2017 [59]. In the absence of new medical interventions, the number of cases will certainly continue to grow.

During its initial discovery approximately half a century ago, all *A. baumannii* isolates were characterized as being a member of either global clone 1 (GC1) or global clone 2 (GC2) families [60,61,62]. These clonal groups were initially known to only possess genes that conferred resistance to tetracycline, sulfonamides, and aminoglycosides but eventually went on to develop resistance to carbapenems, fluoroquinolones, and some third generation cephalosporins, but how and when these resistance traits developed varied between clonal groups. Eventually, there was considerable genetic variation in the genes responsible for the structures of *A. baumannii* lipooligosaccharide (LOS) and capsule, allowing researchers to genetically distinguish between clonal groups and to determine antibiotic resistance [60,63,64,65]. For example, there are a group of GC1 *A. baumannii* strains that became resistant to third generation cephalosporins through the insertion of Tn6168, a ISAba1-bounded transposon that contains a second copy of an *ampC* gene [66]. In addition, another GC1 clade possess a OCL3 variant that alters the structure of LOS compared to other strains that possess the OCL1 variant which conferred carbapenem resistance [63,65]. These variants, along with many others, highlight the extraordinary clonality associated with *A. baumannii* isolates.

*A. baumannii*’s remarkable ability to develop resistance to numerous classes of antibiotics occurs through multiple mechanisms, including the acquisition of mutations in antibiotic targets; modification of the antibiotic itself; decreased outer membrane permeability to limit the entrance of antibiotics, and multi-drug efflux pumps. For example, *A. baumannii* contains an abundance of membrane transporters from the resistance-nodulation-cell superfamily. These transporters are known to contribute to chloramphenicol, macrolide, trimethoprim, fluoroquinolone, aminoglycoside, and β-lactam resistance [67,68]. In addition, numerous reports have documented *A. baumannii* clinical strains with resistance to colistin, an antibiotic typically used as a last resort treatment [69]. This is particularly concerning as the rate of new antibiotics entering the market has steeply declined over the past several decades. This drop off is largely due to an increase in costs necessary to navigate regulatory agency approval processes; the relatively short lifespan of an antibiotic due to rapid development of antibiotic resistance; and the high profit margins associated with pharmaceutical companies switching to developing and manufacturing drugs used for chronic conditions including cancer, heart disease, and persistent viral infections such as HIV and Hepatitis [70].

While select antibiotics retain some effectiveness against *A. baumannii*, including the cephalosporin Cefiderocol and newer tetracyclines like Eravacycline and TP-6076, there is a real threat that emerging antimicrobial resistance will eventually render these novel antibiotics useless [71]. This uphill battle against *A. baumannii* stresses the importance of shifting the medical community’s focus from therapeutics to strategies that can prevent infections in the first place.

## 2. Vaccine Candidates for *A. baumannii*

Vaccination represent a promising and plausible strategy for preventing *A. baumannii* infections. Moreover, the large degree of clonality observed among *A. baumannii* clinical isolates may result in broad vaccine coverage if conserved antigens are targeted. In this regard, a multitude of vaccine candidates have been explored for *A. baumannii* over many decades with varying degrees of success. In the past decade, a number of multivalent vaccines including live attenuated strains, bacterial ghosts, outer membrane vesicles/complexes, and DNA or subunit vaccines have been explored with promising outcomes (Table 1). In this review, we will examine each of these platforms and summarize the immunogenicity and protective efficacy observed in rodent models. To our knowledge, none of the candidates discussed in this review have advanced beyond pre-clinical evaluation.

### 2.1. Live-Attenuated Strains

Live-attenuated vaccines are composed of bacteria that have been altered to reduce or eliminate pathogenicity, allowing the immune system to encounter the pathogen without the risk of disease. The main benefit of using live-attenuated strains for vaccination is that the mutant strain presents multiple antigens that mirror many of those presented by fully virulent strains, producing both antibody and cellular immune responses to multiple targets [72]. The drawbacks of live attenuated vaccines is the possibility of reversion to virulence, either through horizontal gene transfer or spontaneous mutations [72]. It is also unclear if live attenuated vaccines would be approved for use in immunocompromised populations, such as those at greatest risk for *A. baumannii* infection. Few vaccine studies utilizing live attenuated strains of *A. baumannii* have been performed in recent years, but a D-glutamate strain of *A. baumannii* was recently created and evaluated as a live attenuated vaccine [73]. The mutations introduced in the *murI1* and *murI2* genes created a strain of *A. baumannii* that was less virulent in BALB/c mice but still generated both antibody- and cell-mediated immunity and conferred increased survival to immunized animals after challenge with multiple, pathogenic strains of *A. baumannii* [73]. A similar study examined the protection elicited by a thioredoxin-deficient *A. baumannii* strain (Δ*trxA*) [74]. Thioredoxins are crucial for bacteria to counter oxidative stresses and redox regulations. Vaccination with the Δ*trxA* strain led to full protection against infection with a clinical *A. baumannii* isolate [74,75].

### 2.2. Bacterial Ghosts

Bacterial ghosts (BG) begin as live bacteria but are processed to remove all cytosolic components to leave behind only the outer membrane of the bacteria. The use of BGs present a few inherent advantages as they are nonliving and therefore incapable of reverting to a pathogenic phenotype [72]. In addition, BGs are self-adjuvanting and can be modified to carry a unique combination of proteins, DNA, drugs or other small molecules, and have been demonstrated to be efficiently taken up by antigen presenting cells in specific tissues [72]. However, because BGs contain the original bacterial outer membrane, there is some risk of excessive inflammation caused by the presence of native LPS [72]. *A. baumannii* BGs (strain Ali190) were used to vaccinate Sprague-Dawley rats orally, subcutaneously, intramuscularly, or intraperitoneally before being challenged with 10^8^ CFU of the homologous *A. baumannii* strain [76]. *A. baumannii* BGs were highly effective at protecting the vaccinated rats via all routes of administration, with the exception of oral vaccination that conferred only 67% survival [76]. While this initial study yielded positive results, it is imperative to test the ability of *A. baumannii* BGs to protect against a variety of *A. baumannii* strains, especially distinct clades to determine if protection is serotype-dependent or independent for BGs.

### 2.3. Outer Membrane Vesicles or Complexes

Outer membrane vesicles (OMVs) are small, non-infectious particles secreted by Gram-negative bacteria, including *A. baumannii*, that range in size from 10–300 nm in diameter [77]. OMVs are composed of a wide variety of bacterial components, including DNA, RNA, lipo- and polysaccharides, and proteins. As such, OMVs are multivalent in nature and provide multiple antigens that can elicit both antibody and T cell responses against numerous bacterial targets [78]. Previous studies have already validated OMV-based vaccines using a diverse array of bacteria including *Escherichia coli* [79,80,81], *Bordetella pertussis* [82,83], *Burkholderia pseudomallei* [84,85,86,87], and even a commercially available vaccine for *Neisseria meningitidis* serogroup B [88,89].

Multiple studies have evaluated the efficacy of OMV-based *A. baumannii* vaccines. In one study, mice vaccinated in a prime-boost fashion demonstrated increases in overall IgG, IgG1, IgG2, and IgM titers, significantly lower bacterial loads, and a significantly higher survival rate compared to their unvaccinated counterparts [90]. The serum obtained from vaccinated mice recognized many of the proteins predicted to be present in OMVs, including OmpA, CarO, OmpW, and a multitude of other putative outer membrane proteins [90]. In a second study, mice vaccinated with *A. baumannii* OMVs produced anti-*A. baumannii* IgG antibodies and displayed increased sensitivity to levofloxacin in an otherwise resistant *A. baumannii* strain [77]. The exact mechanism behind this observation is unknown, but the authors speculated that the vaccine-induced antibodies may target the porins used to export antibiotics from the bacterial cytoplasm, thus increasing susceptibility to quinolone antibiotics.

A potential concern with any OMV-based vaccine is the role LPS plays in immunogenicity and the potential adverse side effects caused by an abundance of endotoxin. These concerns were addressed, in part, by isolating OMVs from the IB010 *A. baumannii* [91]. The IB010 strain possesses a mutated *lpxD* gene making it incapable of producing LPS [91]. Compared to mice vaccinated with OMVs from the parental ATCC19606 strain, the LPS-negative OMVs elicited a similar IgG1, IgG2a, and IgM responses and mice vaccinated with either OMV type had almost identical bacterial loads [91]. However, the LPS-negative OMVs only protected 75% of the mice challenged with *A. baumannii* compared to complete survival of mice vaccinated with a similar amount of WT OMVs [91]. Mice vaccinated with LPS-negative OMVs were completely protected when the concentration of OMVs used was increased 10-fold per mouse or when combined with exogenous LPS [91]. The same bacterial strain was also used in a study to evaluate protection elicited from outer membrane complexes (OMCs) isolated from LPS- *A. baumannii*. Similar to the LPS- negative OMVs, there was little to no toxicity associated with the LPS-deficient OMCs and mice vaccinated with LPS-deficient OMCs had a 60% survival rate at day 7 of the study compared a 95% survival rate of mice vaccinated with LPS-deficient OMCs supplemented with exogenous LPS [92]. Both groups faired significantly better when compared to the 0% survival of control mice [92]. Results from these studies would suggest that, while the absence of LPS may guard against excessive inflammation, the presence of LPS antigen within an OMV-based vaccine appears to contribute to protection.

Finally, it is well-appreciated that the bacterial culture conditions and OMV purification methods will affect final OMV composition. To evaluate this, three different OMV isolation protocols were compared with surprising results. OMVs harvested from supernatants using a series of ultracentrifugation and filtration steps (sOMVs), OMVs harvested through sonicating bacterial cultures (suOMVs), and OMVs obtained by shearing live bacteria using a high-speed disperator (nOMVs) had appreciably different protein profiles. OmpA was the major protein in all OMVs and each purification method resulted in the presence of LPS [93]. More importantly, however, was the variation in immune responses elicited by each type of OMV. Following vaccination, mice given suOMVs had the highest IgG titers, followed by sOMVs, and nOMVs [93]. Not surprisingly, the survival rates of mice challenged with *A. baumannii* LAC-4 followed the same trend with 70% of mice given suOMVs surviving while only 60%, 50%, and 20% of sOMV vaccinated, nOMV vaccinated, or naive mice surviving, respectively [93]. Collectively, these studies suggest that *Acinetobacter* OMV-based vaccines show promise, but more research is warranted in order to identify which OMV components are critical for vaccine protection.

### 2.4. DNA-Based Vaccines

OmpA has received considerable attention as a vaccine antigen because of its ubiquity across *A. baumannii* clinical isolates and its numerous roles in infection including antimicrobial resistance, biofilm formation, and adherence to host cells [94]. Recently, OmpA was the subject of a DNA-based vaccine that was evaluated in BALB/c mice [94]. The OmpA gene was inserted into the eukaryotic expression pBudCD4.1 vector and injected intramuscularly into mice in a prime-boost fashion [94]. One month after vaccination, IgM and IgG, and the cytokines IL-2, IL-4, IL-12, and IFN-γ levels were significantly increased in vaccinated mice compared to mice vaccinated with an empty vector control [94]. Mice vaccinated with the OmpA DNA vaccine faired considerably better than their negative control counterparts, with 60% of vaccinated mice surviving compared to 0% of the non-immunized mice after intranasal challenge with a lethal dose of *A. baumannii* [94].

A second group expanded on the initial OmpA DNA vaccine by incorporating the *pal* gene into an OmpA DNA-based vaccine [95]. Pal, a lipoprotein found in both the bacterial membrane and peptidoglycan layer, was chosen as a vaccine antigen because of its ubiquity in *A. baumannii* isolates and high level of immunogenicity during natural infection [95]. Both genes were inserted into the pVAX1 expression plasmid and injected into C57BL/6 mice intramuscularly and eventually challenged with *A. baumannii* intratracheally [95]. All unvaccinated mice died within 3 days of the infection while 60% of mice vaccinated with the Pal/OmpA DNA vaccine survived the length of the study and had lower bacterial burdens 24 h after infection compared to other groups [95]. While these two studies support the idea of pursing DNA vaccines, additional research may be necessary to improve delivery, potency, and eventual licensure of DNA-based vaccines.

### 2.5. Purified or Recombinant Subunit Vaccines

Surface or outer membrane antigens of *A. baumannii* represent plausible targets for vaccine-induced opsonizing or bactericidal antibodies. One of the complicating factors in targeting components of the *A. baumannii* outer membrane is the presence of a dense polysaccharide capsule that shields most outer membrane antigens from immune recognition [96]. In fact, monoclonal antibodies raised against the *A. baumannii* K1 capsular polysaccharide were shown to decrease K1-positive strain burdens in mice while having no effect on K1-negative strains, suggesting that immunotherapies using monoclonal antibodies against capsular polysaccharides could be effective against *A. baumannii* infections if clinicians are able to rapidly identify and target the correct capsule profile [97]. Nonetheless, *A. baumannii* does utilize effector proteins to interact with the extracellular environment and a portion of each protein may extend through the capsule where it can be recognized by the immune system. Identifying those proteins and, more specifically, the regions of those proteins that extend into the environment can provide novel vaccine targets. This strategy has been employed for a number of *A. baumannii* virulence determinants, including BAP (biofilm-associated protein), BAP-like protein 1 (Blp1), OmpA, Omp22, and CSU/Pili.

BAP and Blp1 are distinct proteins that contribute to *A. baumannii* biofilm formation and adherence to biotic and abiotic surfaces, and both are necessary to establish infection [98,99]. In one study, Blp1-specific antiserum was raised in mice and subsequently shown to neutralize and promote opsonophagocytic killing of *A. baumannii* pandemic strains (international clonal lineages I (IC I) and II (IC II)) [99]. Mice vaccinated with the C-terminal region of the Blp1 protein had an increased survival rate compared to their unvaccinated counterparts, which suggests immune recognition of the C-terminal region of Blp1 could serve as a starting point for a purified recombinant vaccine against *A. baumannii* [99]. Similar to the Blp1 study, recombinant BAP was used as a vaccine antigen in mice to induce a robust antibody response [100]. In addition to the antibody response, mice vaccinated with recombinant BAP had decreased bacterial burdens and increased survival compared to controls [100]. These studies serve as proof of concept that immunizing with proteins necessary for *A. baumannii* pathogenesis, specifically biofilm formation, may constitute a protective vaccine.

Outer membrane proteins (Omps) are generally conserved across *A. baumannii* isolates and contribute to a multitude of *A. baumannii* traits that complicate treatment including antibiotic resistance, adherence to host cell, and pathogenesis. Omps are found in high abundance within bacterial outer membranes, often protruding through the polysaccharide capsule, making the Omp family of proteins an appropriate vaccine candidate. This has been the case with Oma87 [101], the N-terminal region of Outer membrane porin F (OprF) [102], Omp22 [103], and OmpA [104,105,106]. Each of these outer membrane protein-based vaccines elicited a robust IgG response and increased the survival rates of mice compared to the naïve control mice [101,102,103,104,105,106]. OmpA was combined with the secreted serine protease PKF in an antigen cocktail and evaluated for protection against *A. baumannii* infection [107]. Individually, PKF- and OmpA-immunized animals displayed a 80% and 75% survival rate, respectively, but when combined, OmpA and PKF protected 85% of the mice vaccinated with the duel-component vaccine [107]. Finally, the outer membrane protein FilF, used by *A. baumannii* during the course of pilus assembly, is exposed to the extracellular environment and was evaluated as a vaccine candidate. Vaccination with FilF decreased the bacterial load in the lungs by 4 logs compared to the adjuvant only control; reduced pro-inflammatory cytokine levels; and led to a 50% survival rate relative to the 0% survival rate of control mice [108]. Of course, outer membrane proteins are transmembrane proteins composed of hydrophilic and hydrophobic regions that impact native confirmation. These regions complicate the purification process but can be overcome by including a series of hydrophilic amino acids at the N-terminal region to increase solubility in hydrophilic environments [109]. Many strategies have been developed to purify these complex proteins, but the overall complexity and costs associated with Omp-based vaccines may prove to be difficult.

There are other proteins present in the *A. baumannii* outer membrane outside of the Omp family of proteins, in particular the trimeric autotransporter adhesion (Ata), BamA, NucAb, SmpA, and the components of *A. baumannii* pili. Ata is part of an autotransporter system unique to *A. baumannii* that is used for adherence to eukaryotic cells or extracellular matrix proteins and contributes to virulence [110,111]. When tested in a murine model, recombinant Ata vaccine elicited a robust antibody response that prevented bacterial adherence, while simultaneously driving opsonophagocytic and bactericidal activities and inducing protective immunity [112]. These results, coupled with the knowledge that *ata* is a broadly conserved gene across all *Acinetobacter* species provides support for pursing Ata as a future *A. baumannii* vaccine [112]. The outer membrane assembly factor BamA was similarly identified as a potential vaccine candidate through in silico analysis. The highly conserved protein elicited a high antibody titer and protected 80% of vaccinated mice when challenged with a lethal dose of *A. baumannii* through intranasal challenge [113].

A reverse vaccinology approach identified an outer membrane nuclease designated as NucAb as a potential vaccine target based on its non-homology to host proteins, location within the outer membrane, prevalence among clinical isolates, and the presence of anti-NucAb antibodies in mice challenged with *A. baumannii* [114]. Mice vaccinated with recombinant NucAb had suppressed inflammation levels and reduced pro-inflammatory cytokines, with the majority of lung inflammation in vaccinated mice resolved within 24 h compared to unvaccinated mice that still had an abundance of bacteria present with necrotic exudates and robust inflammation [114]. However, only 20% of the vaccinated mice survived compared to 0% survival of control mice [114]. Another reverse vaccinology paper predicted the phospholipase D (PLD) produced by *A. baumannii*, along with an outer membrane lipoprotein named small protein A (SmpA), could elicit a protective immune response [115]. Mice vaccinated with both SmpA and PLD had the highest survival (66.7%), the lowest levels of pro-inflammatory cytokines, the best histology scores following challenge, and the lowest bacterial loads at 3 days post infection [115].

A common feature of many bacterial pathogens is the presence of pili. *A. baumannii* utilizes its pili for twitching motility, host cell adherence, and natural competence [116]. Components of pili are exposed to the extracellular environment and can be detected by the immune system. Two pilus *A. baumannii* proteins, CsuA/B and FimA, are known to be recognized by the host’s immune system during infection and stimulate antibody responses. Vaccination with either protein elicited a robust antibody titer in BALB/c mice and, when used together in a vaccine cocktail, produced a 60% survival rate compared to FimA (50%) or CsuA/B (35%) given alone [117].

Another group of vaccine candidates constitute elements used for nutrient scavenging by *A. baumannii.* Numerous groups have hypothesized that blocking bacterial acquisition of iron or zinc during infection could limit or prevent disease. ZnuD is a zinc outer membrane receptor that contains four loops that are exposed to the extracellular environment and is necessary for *A. baumannii* survival in the zinc-depleted environments inside host cells [118]. One study assessed vaccination of mice with recombinant proteins containing one or all ZnuD loops. The loops were attached to the C-lobe of the surface lipoprotein TbpB which served as a scaffold during the purification process [118]. The presence of the TbpB scaffold provided no protection against *A. baumannii* in the mouse model, but the addition of any one loop led to high antibody titers and 25–50% survival in vaccinated mice. However, 100% of the mice survived an *A. baumannii* challenge when all four of the ZnuD loops were added to the TbpB scaffold [118]. While the production of such a recombinant protein on a commercial scale could by costly due to the intricacies of protein loop folding, these studies do indicate that specific residues of surface-exposed *A. baumannii* factors can lead to recombinant vaccines that provide high levels of protection in animal models.

Finally, it has been shown that high levels of homology across bacterial genera can lead to cross-protective vaccines that protect against multiple bacterial genera. As an example, it was shown that vaccination against *Mycobacterium tuberculosis* using Bacillus Calmette-Guérin can protect against *Staphylococcus aureus* [119] and that a live recombinant attenuated *Salmonella* can protect against a *Mycobacterium tuberculosis* infection [120]. It is less appreciated, however, that homology extends to other animal kingdoms, specifically fungi. A computation modeling study identified a number of similarities between the *Candida albicans* hyphal wall protein Hyr1p and multiple *A. baumannii* cell surface proteins including hemagglutinin (FhaB) and OmpA [121]. Vaccination with Hyr1p led to a 50% survival rate compared to mice vaccinated with adjuvant alone, all of which died 10 days post infection [121]. This study highlights the potential for AI and computational models to design vaccines that may provide protection against not only multiple bacterial genera, but across different kingdoms of pathogens.

## 3. Conclusions

*A. baumannii* will likely remain a difficult organism to treat clinically due to its remarkable ability to develop antimicrobial resistance. However, a growing number of studies suggest that an effective vaccine could be developed against this organism. Several of the vaccine studies summarized in this review utilized computer learning and prediction software, and vaccine success in these pre-clinical studies indicates that AI and computer modeling should help inform rationale vaccine design in the future. Going forward, it will be important to identify which protective antigens are most highly conserved among *A. baumannii* clinical isolates in order to formulate a broadly protective multivalent vaccine. While some monovalent subunit vaccines successfully protected mice challenged with various clinical strains of *A. baumannii*, it is highly likely that multiple antigens will be necessary for complete vaccine protection. Analysis of the *A. baumannii* vaccine studies discussed in this review reveal the most effective vaccines are multivalent in nature (composed of outer membrane vesicles, bacterial ghosts, or multi-subunit) and are often composed of antigens present on the outer membrane of the bacteria (OmpA, OmpW, OmpK, and Omp22) [73,91,103,122,123,124,125,126,127,128]. This observation suggests that a cocktail of conserved surface proteins could be the most effective vaccine against a majority of *A. baumannii* clinical strains and warrants further investigation. This is not particularly surprising given that there are many FDA approved multicomponent vaccines on the market today, including the Diphtheria, Tetanus, and Pertussis (DTaP) and Meningitis B (MenB) vaccines. Lessons from other opportunistic bacteria, such as *Streptococcus pneumoniae*, highlight the importance of antigen choice to avoid the development of vaccine escape strains. In addition, the target population will influence the type of vaccine that is required as demonstrated by capsular polysaccharide-based vaccines for the elderly versus conjugate vaccines for children to prevent infection with pneumococcus. In this regard, it is important to determine which target populations require an *A. baumannii* vaccine. Although nosocomial infections with *A. baumannii* are worrisome, it is difficult to predict whether an individual will become hospitalized and require such a vaccine. Vaccination of healthcare workers could potentially limit the abundance and/or spread of *A. baumannii* in the hospital setting, but this would require a vaccine that eliminates bacterial colonization and carriage and may be difficult to accomplish. In the short term, populations that are most at risk include members of the armed forces. A vaccine designed to immunize military personnel must display an excellent safety profile, with minimal to no reactogenicity, in order to preserve military readiness. In the long term, the antimicrobial resistance crisis may dictate that all individuals require vaccination against *A. baumannii*, particularly if *A. baumannii* develops complete drug-resistance and becomes untreatable. 

## Figures and Tables

**Table 1:** Representative list of *A. baumannii* vaccine studies conducted over the past decade reported in NCBI.

Immunization	Challenge								
Vaccine Platform	Antigens	Adjuvant(s)	Route	Mouse Strain	Strain	Route	Dose	Survival	Ref
Whole bacteria	*A. baumannii* Δ*murl1* or Δ*murl2*	-	IP	BALB/c	ATCC 17978	IP	3 × 10^8^ CFU	100%	[73]
	*A. baumannii* Δ*trxA*	-	IP, SC	C57BL/6	Clinical Isolate 79 (Ci79)	IP	10^4^–10^7^ CFU	IP—100% SC—90%	[74]
	*A. baumannii* (Ali190) BGs	Freund’s adjuvant	Oral, SC, IM, IP, SCA, IMA	Sprague-Dawley rats (male)	Ali190	IP	10^8^ CFU	Oral—67% SC, IM, IP, SCA, IMA—100%	[76]
	Formalin-killed *A. baumannii* LAC-4 cells (5 × 10^7^ CFU)	-	IN	WT, Igh-Jtm1Dhu, and Fcer1gtm1Rav BALB/c	LAC 4	IN or IP	5 × 10^7^ CFU	IN challenge—100% IP challenge—100%	[122]
Outer membrane vesicles or complexes	OMVs isolated from *A. baumannii* strain 19606	Alum	SC	C57BL/6	Ab112	IP	10^7^ CFU	OMV vaccine w/Levofloxacin—85% OMV vaccine only—0%	[77]
	OMVs isolated from *A. baumannii* strain 19606	Alum	IM	C57BL/6	ATCC 19606, Ab-154, Ab-113-16	IP	4.5 × 10^5^	19606 challenge—100% Ab-154 challenge—90% Ab-113-16—100%	[90]
	LPS-negative OMVs isolated from IB010	Alum	IM	C57BL/6	ATCC 19606	IP	1.8 × 10^6^ CFU	70%	[91]
	OMCs isolated from *A. baumannii* strain IB010 (Δ*lpxD*)	Alum	IM	C57BL/6	ATCC 19606	IP	1.6 × 10^6^	60%	[92]
	SuOMVs, nOMVs, or sOMVs isolated from strain 17978	Alum	IM and IN	C57BL/6	LAC 4	IT	2 × 10^7^ CFU	IM—60–100% IN—50–70%	[93]
	OMVs isolated from DH5α *E.coli* displaying *A. baumannii* Omp22	Alum	SC	ICR	Ab1	IP	1.6 × 10^6^	100%	[123]
	*A. baumannii* IB010	Alum	IM	C57BL/6	ATCC 19606	IP	10^8^ CFU	100%	[124]
	OMVs isolated from *A. baumannii* strain Ab1	Alum	IM	ICR	Ab1	IN or IP	IN challenge—5 × 10^7^ IP challenge—5.5 × 10^5^	IN challenge—100% IP challenge—73%	[125]
DNA-based vaccine	DNA encoding *A. baumannii* OmpA gene	Alum	IM	BALB/c	MDR clinical *A. baumannii* strain	IN	10^8^ CFU	60%	[94]
	DNA encoding genes for *A. baumannii* OmpA and Pal	CpG	IM	C57BL/6	LAC 4	IN or IT	3 × 10^7^ CFU	IN challenge—80% IT Challenge—50%	[95]
Purified or Recombinant Subunit	rHis-Blp1_(2652–3362)_	Freund’s Adjuvant	IM	BALB/c	Ab1	IP	10^8^ CFU	60%	[99]
	rHis-Bap	Freund’s Adjuvant	IM	BALB/c	Clinical isolate	IP	10^8^ CFU	80%	[100]
	rHis-Oma87	Freund’s Adjuvant	SC	BALB/c	19606	IP	2 × 10^6^ CFU	100%	[101]
	rHis-OprF_(25–200)_	BCG and Alum	SC	Swiss Albino	9027	IP	3.2 × 10^9^ CFU	50%	[102]
	rHis-Omp22	Alum	SC	ICR	17978	IP	1.6 × 10^6^ CFU	100%	[103]
	rHis-OmpA	Alum	SC	Retired breeder or juvenile BALB/c	HUMC1	IV	2 × 10^7^ CFU	Retired breeder—50% Juvenile—45%	[104]
	rHis-OmpA	Cholera toxin	IN	BALB/c	19606	IP	5 × 10^8^ CFU	50%	[106]
	rHis-PKF with anti- *A. baumannii* OmpA antibodies	Alum	IP	C57BL/6	19606	IP	10^8^ CFU	85.71%	[107]
	rHis-FilF	Freund’s Incomplete Adjuvant	SC	BALB/c	19606	IT	10^8^ CFU	50%	[108]
	rHis-BamA	Alum	SC	BALB/c	19606	IN	10^9^ CFU	70%	[113]
	rHis-NucAb	Freund’s complete adjuvant	IP	BALB/c	19606	IT	10^8^ CFU	20%	[114]
	rHis-SmpA/PLD	Alum	SC	BALB/c	ST191	IT	10^8^ CFU	66.7%	[115]
	rHis-CsuA/B and rHis-FimA	Freund’s Complete Adjuvant	SC	BALB/c	19606	IP	2 × 10^8^ CFU	Csu-Fim—60% Fim—50% Csu—33.3%	[117]
	rHis-ZnuD (loop 2, 5, 7, or 11) attached to TbpA scaffold	Freund’s Complete Adjuvant	SC	BALB/c	19606	IP	10^7^ CFU	Loop 2—40% Loop 5—30% Loop 7—50% Loop 11—35% Loop 2,5,7,11—100%	[118]
	rHyr1p-N (*Candida albicans*)	Alum	SC	BALB/c	HUMC1	Aerosol	5 × 10^7^ CFU	50%	[121]
	rHis-OmpK/Omp22	MF59	IT	BALB/c	19606	IT	10^8^ CFU	83.3%	[126]
	rHis-OmpK/Omp22	Freund’s complete adjuvant	SC	BALB/c	Ab1	IP	2 x 10^8^ CFU	66.7%	[127]
	rHis-OmpW	Alum	SC	ICR	Ab1	IP	10^6^ CFU	100%	[128]

BGs = Bacterial ghost. SC = subcutaneous. SCA = subcutaneous with adjuvant. IMA = intramuscular with adjuvant. IT = Intratracheal. IP = Intraperitoneal. IM = Intramuscular. IN = Intranasal. IV—Intravenous. Aerosol = Aerosolization.

## References

[1] Fernando D.M., Khan I.U.H., Patidar R., Lapen D.R., Talbot G., Topp E., Kumar A. (2016). Isolation and Characterization of *Acinetobacter baumannii* Recovered from Campylobacter Selective Medium. Front. Microbiol..

[2] Harding C.M., Hennon S.W., Feldman M.F. (2018). Uncovering the mechanisms of *Acinetobacter baumannii* virulence. Nat. Rev. Microbiol..

[3] Fitzpatrick M.A., Ozer E.A., Hauser A.R. (2016). Utility of Whole-Genome Sequencing in Characterizing *Acinetobacter* Epidemiology and Analyzing Hospital Outbreaks. J. Clin. Microbiol..

[4] Sheppard F.R., Keiser P., Craft D.W., Gage F., Robson M., Brown T.S., Petersen K., Sincock S., Kasper M., Hawksworth J. (2010). The majority of US combat casualty soft-tissue wounds are not infected or colonized upon arrival or during treatment at a continental US military medical facility. Am. J. Surg..

[5] Centers for Disease Control and Prevention (CDC) (2004). *Acinetobacter baumannii* infections among patients at military medical facilities treating injured U.S. service members, 2002–2004. MMWR Morb. Mortal. Wkly. Rep..

[6] Scott P., Deye G., Srinivasan A., Murray C., Moran K., Hulten E., Fishbain J., Craft D., Riddell S., Lindler L. (2007). An outbreak of multidrug-resistant *Acinetobacter baumannii*-*calcoaceticus* complex infection in the US military health care system associated with military operations in Iraq. Clin. Infect. Dis..

[7] Lindberg R.B., Wetzler T.F., Marshall J.D., Newton A., Strawitz J.G., Howard J.M. (1955). The bacterial flora of battle wounds at the time of primary debridement; a study of the Korean battle casualty. Ann. Surg..

[8] Murray C.K., Yun H.C., Griffith M.E., Hospenthal D.R., Tong M.J. (2006). *Acinetobacter* infection: What was the true impact during the Vietnam conflict?. Clin. Infect. Dis..

[9] Hawley J.S., Murray C.K., Griffith M.E., McElmeel M.L., Fulcher L.C., Hospenthal D.R., Jorgensen J.H. (2007). Susceptibility of *Acinetobacter* strains isolated from deployed U.S. military personnel. Antimicrob. Agents Chemother..

[10] Towner K.J. (2009). *Acinetobacter*: An old friend, but a new enemy. J. Hosp. Infect..

[11] Morris F.C., Dexter C., Kostoulias X., Uddin M.I., Peleg A.Y. (2019). The Mechanisms of Disease Caused by *Acinetobacter baumannii*. Front. Microbiol..

[12] Chung D.R., Song J.-H., Kim S.H., Thamlikitkul V., Huang S.-G., Wang H., So T.M., Yasin R.M.D., Hsueh P.-R., Carlos C.C. (2011). High Prevalence of Multidrug-Resistant Nonfermenters in Hospital-acquired Pneumonia in Asia. Am. J. Respir. Crit. Care Med..

[13] Dananché C., Vanhems P., Machut A., Aupée M., Bervas C., L’Hériteau F., Lepape A., Lucet J.-C., Stoeckel V., Timsit J.-F. (2018). Trends of Incidence and Risk Factors of Ventilator-Associated Pneumonia in Elderly Patients Admitted to French ICUs between 2007 and 2014. Crit. Care Med..

[14] Tabah A., Koulenti D., Laupland K., Misset B., Valles J., Bruzzi de Carvalho F., Paiva J.A., Çakar N., Ma X., Eggimann P. (2012). Characteristics and determinants of outcome of hospital-acquired bloodstream infections in intensive care units: The EUROBACT International Cohort Study. Intensive Care Med..

[15] Garnacho-Montero J., Timsit J.-F. (2019). Managing *Acinetobacter baumannii* infections. Curr. Opin. Infect. Dis..

[16] Ayoub Moubareck C., Hammoudi Halat D. (2020). Insights into *Acinetobacter baumannii*: A Review of Microbiological, Virulence, and Resistance Traits in a Threatening Nosocomial Pathogen. Antibiotics.

[17] Griffith M.E., Lazarus D.R., Mann P.B., Boger J.A., Hospenthal D.R., Murray C.K. (2007). *Acinetobacter* skin carriage among US army soldiers deployed in Iraq. Infect. Control Hosp. Epidemiol..

[18] Hujer K.M., Hujer A.M., Hulten E.A., Bajaksouzian S., Adams J.M., Donskey C.J., Ecker D.J., Massire C., Eshoo M.W., Sampath R. (2006). Analysis of antibiotic resistance genes in multidrug-resistant *Acinetobacter* sp. isolates from military and civilian patients treated at the Walter Reed Army Medical Center. Antimicrob. Agents Chemother..

[19] Mohd Sazlly Lim S., Zainal Abidin A., Liew S.M., Roberts J.A., Sime F.B. (2019). The global prevalence of multidrug-resistance among *Acinetobacter baumannii* causing hospital-acquired and ventilator-associated pneumonia and its associated mortality: A systematic review and meta-analysis. J. Infect..

[20] Chen N., Zhou M., Dong X., Qu J., Gong F., Han Y., Qiu Y., Wang J., Liu Y., Wei Y. (2020). Epidemiological and clinical characteristics of 99 cases of 2019 novel coronavirus pneumonia in Wuhan, China: A descriptive study. Lancet.

[21] Rawson T.M., Moore L.S.P., Zhu N., Ranganathan N., Skolimowska K., Gilchrist M., Satta G., Cooke G., Holmes A. (2020). Bacterial and Fungal Coinfection in Individuals With Coronavirus: A Rapid Review To Support COVID-19 Antimicrobial Prescribing. Clin. Infect. Dis..

[22] Lai C.-C., Wang C.-Y., Hsueh P.-R. (2020). Co-infections among patients with COVID-19: The need for combination therapy with non-anti-SARS-CoV-2 agents?. J. Microbiol. Immunol. Infect..

[23] Yu N., Li W., Kang Q., Xiong Z., Wang S., Lin X., Liu Y., Xiao J., Liu H., Deng D. (2020). Clinical features and obstetric and neonatal outcomes of pregnant patients with COVID-19 in Wuhan, China: A retrospective, single-centre, descriptive study. Lancet Infect. Dis..

[24] Wang Z., Yang B., Li Q., Wen L., Zhang R. (2020). Clinical Features of 69 Cases With Coronavirus Disease 2019 in Wuhan, China. Clin. Infect. Dis..

[25] Zhu X., Ge Y., Wu T., Zhao K., Chen Y., Wu B., Zhu F., Zhu B., Cui L. (2020). Co-infection with respiratory pathogens among COVID-2019 cases. Virus Res..

[26] Lescure F.-X., Bouadma L., Nguyen D., Parisey M., Wicky P.-H., Behillil S., Gaymard A., Bouscambert-Duchamp M., Donati F., Le Hingrat Q. (2020). Clinical and virological data of the first cases of COVID-19 in Europe: A case series. Lancet Infect. Dis..

[27] Fan J., Li X., Gao Y., Zhou J., Wang S., Huang B., Wu J., Cao Q., Chen Y., Wang Z. (2020). The lung tissue microbiota features of 20 deceased patients with COVID-19. J. Infect..

[28] Lima W.G., Brito J.C.M., da Cruz Nizer W.S. (2020). Ventilator-associated pneumonia (VAP) caused by carbapenem-resistant *Acinetobacter baumannii* in patients with COVID-19: Two problems, one solution?. Med. Hypotheses.

[29] Zilberberg M.D., Nathanson B.H., Sulham K., Fan W., Shorr A.F. (2016). Multidrug resistance, inappropriate empiric therapy, and hospital mortality in *Acinetobacter baumannii* pneumonia and sepsis. Crit. Care.

[30] Zilberberg M.D., Nathanson B.H., Sulham K., Fan W., Shorr A.F. (2017). Daily cost of delay to adequate antibiotic treatment among patients surviving a hospitalization with community-onset *Acinetobacter baumannii* pneumonia or sepsis. Crit. Care.

[31] Zurawski D.V., Banerjee J., Alamneh Y.A., Shearer J.P., Demons S.T. (2019). Skin and Soft Tissue Models for *Acinetobacter baumannii* Infection. Methods Mol. Biol..

[32] Tiwari V., Meena K., Tiwari M. (2018). Differential anti-microbial secondary metabolites in different ESKAPE pathogens explain their adaptation in the hospital setup. Infect. Genet. Evol..

[33] Zurawski D.V., Black C.C., Alamneh Y.A., Biggemann L., Banerjee J., Thompson M.G., Wise M.C., Honnold C.L., Kim R.K., Paranavitana C. (2019). A Porcine Wound Model of *Acinetobacter baumannii* Infection. Adv. Wound Care.

[34] Almasaudi S.B. (2018). *Acinetobacter* spp. as nosocomial pathogens: Epidemiology and resistance features. Saudi J. Biol. Sci..

[35] Karaiskos I., Galani L., Baziaka F., Katsouda E., Ioannidis I., Andreou A., Paskalis H., Giamarellou H. (2013). Successful treatment of extensively drug-resistant *Acinetobacter baumannii* ventriculitis and meningitis with intraventricular colistin after application of a loading dose: A case series. Int. J. Antimicrob. Agents.

[36] Jiménez-Mejías M.E., Pachón J., Becerril B., Palomino-Nicás J., Rodríguez-Cobacho A., Revuelta M. (1997). Treatment of multidrug-resistant *Acinetobacter baumannii* meningitis with ampicillin/sulbactam. Clin. Infect. Dis..

[37] Cascio A., Conti A., Sinardi L., Iaria C., Angileri F.F., Stassi G., David T., Versaci A., Iaria M., David A. (2010). Post-neurosurgical multidrug-resistant *Acinetobacter baumannii* meningitis successfully treated with intrathecal colistin. A new case and a systematic review of the literature. Int. J. Infect. Dis..

[38] Karakuzu Z., Iscimen R., Akalin H., Kelebek Girgin N., Kahveci F., Sinirtas M. (2018). Prognostic Risk Factors in Ventilator-Associated Pneumonia. Med. Sci. Monit..

[39] Spellberg B., Bonomo R.A. (2013). “Airborne Assault”: A New Dimension in *Acinetobacter baumannii* Transmission. Crit. Care Med..

[40] Mousa M., Schwartz D., Carmeli Y., Nutman A. (2019). Droplet aerosol dissemination of carbapenem-resistant *Acinetobacter baumannii* surrounding ventilated patients. Infect. Control Hosp. Epidemiol..

[41] Di Venanzio G., Flores-Mireles A.L., Calix J.J., Haurat M.F., Scott N.E., Palmer L.D., Potter R.F., Hibbing M.E., Friedman L., Wang B. (2019). Urinary tract colonization is enhanced by a plasmid that regulates uropathogenic *Acinetobacter baumannii* chromosomal genes. Nat. Commun..

[42] Dijkshoorn L., van Aken E., Shunburne L., van der Reijden T.J.K., Bernards A.T., Nemec A., Towner K.J. (2005). Prevalence of *Acinetobacter baumannii* and other *Acinetobacter* spp. in faecal samples from non-hospitalised individuals. Clin. Microbiol. Infect..

[43] Guerrero D.M., Perez F., Conger N.G., Solomkin J.S., Adams M.D., Rather P.N., Bonomo R.A. (2010). *Acinetobacter baumannii*-Associated Skin and Soft Tissue Infections: Recognizing a Broadening Spectrum of Disease. Surg. Infect..

[44] Thom K.A., Rock C., Jackson S.S., Johnson J.K., Srinivasan A., Magder L.S., Roghmann M.-C., Bonomo R.A., Harris A.D. (2017). Factors Leading to Transmission Risk of *Acinetobacter baumannii*. Crit. Care Med..

[45] Takoi H., Fujita K., Hyodo H., Matsumoto M., Otani S., Gorai M., Mano Y., Saito Y., Seike M., Furuya N. (2019). *Acinetobacter baumannii* can be transferred from contaminated nitrile examination gloves to polypropylene plastic surfaces. Am. J. Infect. Control.

[46] Chapartegui-González I., Lázaro-Díez M., Bravo Z., Navas J., Icardo J.M., Ramos-Vivas J. (2018). *Acinetobacter baumannii* maintains its virulence after long-time starvation. PLoS ONE.

[47] Jawad A., Seifert H., Snelling A.M., Heritage J., Hawkey P.M. (1998). Survival of *Acinetobacter baumannii* on dry surfaces: Comparison of outbreak and sporadic isolates. J. Clin. Microbiol..

[48] Boll J.M., Tucker A.T., Klein D.R., Beltran A.M., Brodbelt J.S., Davies B.W., Trent M.S. (2015). Reinforcing Lipid A Acylation on the Cell Surface of *Acinetobacter baumannii* Promotes Cationic Antimicrobial Peptide Resistance and Desiccation Survival. mBio.

[49] Singh J.K., Adams F.G., Brown M.H. (2018). Diversity and Function of Capsular Polysaccharide in *Acinetobacter baumannii*. Front. Microbiol..

[50] Greene C., Wu J., Rickard A.H., Xi C. (2016). Evaluation of the ability of *Acinetobacter baumannii* to form biofilms on six different biomedical relevant surfaces. Lett. Appl. Microbiol..

[51] Tipton K.A., Chin C.-Y., Farokhyfar M., Weiss D.S., Rather P.N. (2018). Role of Capsule in Resistance to Disinfectants, Host Antimicrobials, and Desiccation in *Acinetobacter baumannii*. Antimicrob. Agents Chemother..

[52] Pakharukova N., Tuittila M., Paavilainen S., Malmi H., Parilova O., Teneberg S., Knight S.D., Zavialov A.V. (2018). Structural basis for *Acinetobacter baumannii* biofilm formation. Proc. Natl. Acad. Sci. USA.

[53] Espinal P., Martí S., Vila J. (2012). Effect of biofilm formation on the survival of *Acinetobacter baumannii* on dry surfaces. J. Hosp. Infect..

[54] Selasi G.N., Nicholas A., Jeon H., Na S.H., Kwon H.I., Kim Y.J., Heo S.T., Oh M.H., Lee J.C. (2016). Differences in Biofilm Mass, Expression of Biofilm-Associated Genes, and Resistance to Desiccation between Epidemic and Sporadic Clones of Carbapenem-Resistant *Acinetobacter baumannii* Sequence Type 191. PLoS ONE.

[55] Chiang S.-R., Jung F., Tang H.-J., Chen C.-H., Chen C.-C., Chou H.-Y., Chuang Y.-C. (2018). Desiccation and ethanol resistances of multidrug resistant *Acinetobacter baumannii* embedded in biofilm: The favorable antiseptic efficacy of combination chlorhexidine gluconate and ethanol. J. Microbiol. Immunol. Infect..

[56] Kasimova A.A., Shneider M.M., Arbatsky N.P., Popova A.V., Shashkov A.S., Miroshnikov K.A., Balaji V., Biswas I., Knirel Y.A. (2017). Structure and Gene Cluster of the K93 Capsular Polysaccharide of *Acinetobacter baumannii* B11911 Containing 5-N-Acetyl-7-N-[(R)-3-hydroxybutanoyl]pseudaminic Acid. Biochemistry (Moscow).

[57] Kenyon J.J., Speciale I., Hall R.M., De Castro C. (2016). Structure of repeating unit of the capsular polysaccharide from *Acinetobacter baumannii* D78 and assignment of the K4 gene cluster. Carbohydr. Res..

[58] Aranda J., Bardina C., Beceiro A., Rumbo S., Cabral M.P., Barbé J., Bou G. (2011). *Acinetobacter baumannii* RecA protein in repair of DNA damage, antimicrobial resistance, general stress response, and virulence. J. Bacteriol..

[59] Centers for Disease Control and Prevention (U.S.) (2019). Antibiotic Resistance Threats in the United States, 2019.

[60] Holt K., Kenyon J.J., Hamidian M., Schultz M.B., Pickard D.J., Dougan G., Hall R. (2016). Five decades of genome evolution in the globally distributed, extensively antibiotic-resistant *Acinetobacter baumannii* global clone 1. Microb. Genom..

[61] Schultz M.B., Pham Thanh D., Tran Do Hoan N., Wick R.R., Ingle D.J., Hawkey J., Edwards D.J., Kenyon J.J., Phu Huong Lan N., Campbell J.I. (2016). Repeated local emergence of carbapenem-resistant *Acinetobacter baumannii* in a single hospital ward. Microb. Genom..

[62] Wright M.S., Haft D.H., Harkins D.M., Perez F., Hujer K.M., Bajaksouzian S., Benard M.F., Jacobs M.R., Bonomo R.A., Adams M.D. (2014). New Insights into Dissemination and Variation of the Health Care-Associated Pathogen *Acinetobacter baumannii* from Genomic Analysis. mBio.

[63] Kenyon J.J., Hall R.M. (2013). Variation in the complex carbohydrate biosynthesis loci of *Acinetobacter baumannii* genomes. PLoS ONE.

[64] Kenyon J.J., Holt K.E., Pickard D., Dougan G., Hall R.M. (2014). Insertions in the OCL1 locus of *Acinetobacter baumannii* lead to shortened lipooligosaccharides. Res. Microbiol..

[65] Kenyon J.J., Nigro S.J., Hall R.M. (2014). Variation in the OC locus of *Acinetobacter baumannii* genomes predicts extensive structural diversity in the lipooligosaccharide. PLoS ONE.

[66] Hamidian M., Hall R.M. (2014). Tn6168, a transposon carrying an ISAba1-activated ampC gene and conferring cephalosporin resistance in *Acinetobacter baumannii*. J. Antimicrob. Chemother..

[67] Damier-Piolle L., Magnet S., Brémont S., Lambert T., Courvalin P. (2008). AdeIJK, a resistance-nodulation-cell division pump effluxing multiple antibiotics in *Acinetobacter baumannii*. Antimicrob. Agents Chemother..

[68] Coyne S., Courvalin P., Périchon B. (2011). Efflux-Mediated Antibiotic Resistance in *Acinetobacter* spp.. Antimicrob. Agents Chemother..

[69] Sun J., Zhang H., Liu Y.-H., Feng Y. (2018). Towards Understanding MCR-like Colistin Resistance. Trends Microbiol..

[70] Årdal C., Balasegaram M., Laxminarayan R., McAdams D., Outterson K., Rex J.H., Sumpradit N. (2020). Antibiotic development—Economic, regulatory and societal challenges. Nat. Rev. Microbiol..

[71] Isler B., Doi Y., Bonomo R.A., Paterson D.L. (2018). New Treatment Options against Carbapenem-Resistant *Acinetobacter baumannii* Infections. Antimicrob. Agents Chemother..

[72] Tabrizi C.A., Walcher P., Mayr U.B., Stiedl T., Binder M., McGrath J., Lubitz W. (2004). Bacterial ghosts – biological particles as delivery systems for antigens, nucleic acids and drugs. Curr. Opin. Biotechnol..

[73] Cabral M.P., García P., Beceiro A., Rumbo C., Pérez A., Moscoso M., Bou G. (2017). Design of live attenuated bacterial vaccines based on D-glutamate auxotrophy. Nat. Commun..

[74] Ainsworth S., Ketter P.M., Yu J.-J., Grimm R.C., May H.C., Cap A.P., Chambers J.P., Guentzel M.N., Arulanandam B.P. (2017). Vaccination with a live attenuated *Acinetobacter baumannii* deficient in thioredoxin provides protection against systemic *Acinetobacter* infection. Vaccine.

[75] Zeller T., Klug G. (2006). Thioredoxins in bacteria: Functions in oxidative stress response and regulation of thioredoxin genes. Naturwissenschaften.

[76] Sheweita S.A., Batah A.M., Ghazy A.A., Hussein A., Amara A.A. (2019). A new strain of *Acinetobacter baumannii* and characterization of its ghost as a candidate vaccine. J. Infect. Public Health.

[77] Huang W., Zhang Q., Li W., Chen Y., Shu C., Li Q., Zhou J., Ye C., Bai H., Sun W. (2019). Anti-outer Membrane Vesicle Antibodies Increase Antibiotic Sensitivity of Pan-Drug-Resistant *Acinetobacter baumannii*. Front. Microbiol..

[78] Mancini F., Rossi O., Necchi F., Micoli F. (2020). OMV Vaccines and the Role of TLR Agonists in Immune Response. IJMS.

[79] Roy K., Hamilton D.J., Munson G.P., Fleckenstein J.M. (2011). Outer Membrane Vesicles Induce Immune Responses to Virulence Proteins and Protect against Colonization by Enterotoxigenic *Escherichia coli*. Clin. Vaccine Immunol..

[80] Kim O.Y., Hong B.S., Park K.-S., Yoon Y.J., Choi S.J., Lee W.H., Roh T.-Y., Lötvall J., Kim Y.-K., Gho Y.S. (2013). Immunization with *Escherichia coli* Outer Membrane Vesicles Protects Bacteria-Induced Lethality via Th1 and Th17 Cell Responses. J. Immunol..

[81] Wang H., Liang K., Kong Q., Liu Q. (2019). Immunization with outer membrane vesicles of avian pathogenic *Escherichia coli* O78 induces protective immunity in chickens. Vet. Microbiol..

[82] Gasperini G., Biagini M., Arato V., Gianfaldoni C., Vadi A., Norais N., Bensi G., Delany I., Pizza M., Aricò B. (2018). Outer Membrane Vesicles (OMV)-based and Proteomics-driven Antigen Selection Identifies Novel Factors Contributing to *Bordetella pertussis* Adhesion to Epithelial Cells. Mol. Cell. Proteom..

[83] Asensio C.J.A., Gaillard M.E., Moreno G., Bottero D., Zurita E., Rumbo M., van der Ley P., van der Ark A., Hozbor D. (2011). Outer membrane vesicles obtained from *Bordetella pertussis* Tohama expressing the lipid A deacylase PagL as a novel acellular vaccine candidate. Vaccine.

[84] Nieves W., Petersen H., Judy B.M., Blumentritt C.A., Russell-Lodrigue K., Roy C.J., Torres A.G., Morici L.A. (2014). A *Burkholderia pseudomallei* Outer Membrane Vesicle Vaccine Provides Protection against Lethal Sepsis. Clin. Vaccine Immunol..

[85] Baker S., Davitt C., Motyka N., Kikendall N., Russell-Lodrigue K., Roy C., Morici L. (2017). A *Burkholderia pseudomallei* Outer Membrane Vesicle Vaccine Provides Cross Protection against Inhalational Glanders in Mice and Non-Human Primates. Vaccines.

[86] Nieves W., Asakrah S., Qazi O., Brown K.A., Kurtz J., AuCoin D.P., McLachlan J.B., Roy C.J., Morici L.A. (2011). A naturally derived outer-membrane vesicle vaccine protects against lethal pulmonary *Burkholderia pseudomallei* infection. Vaccine.

[87] Petersen H., Nieves W., Russell-Lodrigue K., Roy C.J., Morici L.A. (2014). Evaluation of a *Burkholderia Pseudomallei* Outer Membrane Vesicle Vaccine in Nonhuman Primates. Procedia Vaccinol..

[88] Van de Waterbeemd B., Streefland M., van der Ley P., Zomer B., van Dijken H., Martens D., Wijffels R., van der Pol L. (2010). Improved OMV vaccine against *Neisseria meningitidis* using genetically engineered strains and a detergent-free purification process. Vaccine.

[89] Oster P., Lennon D., Ohallahan J., Mulholland K., Reid S., Martin D. (2005). MeNZB?: A safe and highly immunogenic tailor-made vaccine against the New Zealand serogroup B disease epidemic strain. Vaccine.

[90] McConnell M.J., Rumbo C., Bou G., Pachón J. (2011). Outer membrane vesicles as an acellular vaccine against *Acinetobacter baumannii*. Vaccine.

[91] Pulido M.R., García-Quintanilla M., Pachón J., McConnell M.J. (2020). A lipopolysaccharide-free outer membrane vesicle vaccine protects against *Acinetobacter baumannii* infection. Vaccine.

[92] Pulido M.R., García-Quintanilla M., Pachón J., McConnell M.J. (2018). Immunization with lipopolysaccharide-free outer membrane complexes protects against *Acinetobacter baumannii* infection. Vaccine.

[93] Li S., Chen D.-Q., Ji L., Sun S., Jin Z., Jin Z.-L., Sun H.-W., Zeng H., Zhang W.-J., Lu D.-S. (2020). Development of Different Methods for Preparing *Acinetobacter baumannii* Outer Membrane Vesicles Vaccine: Impact of Preparation Method on Protective Efficacy. Front. Immunol..

[94] Ansari H., Tahmasebi-Birgani M., Bijanzadeh M., Doosti A., Kargar M. (2019). Study of the immunogenicity of outer membrane protein A (ompA) gene from *Acinetobacter baumannii* as DNA vaccine candidate in vivo. Iran. J. Basic Med. Sci..

[95] Lei L., Yang F., Zou J., Jing H., Zhang J., Xu W., Zou Q., Zhang J., Wang X. (2019). DNA vaccine encoding OmpA and Pal from *Acinetobacter baumannii* efficiently protects mice against pulmonary infection. Mol. Biol. Rep..

[96] Wang-Lin S.X., Olson R., Beanan J.M., MacDonald U., Balthasar J.P., Russo T.A. (2017). The Capsular Polysaccharide of *Acinetobacter baumannii* Is an Obstacle for Therapeutic Passive Immunization Strategies. Infect. Immun..

[97] Russo T.A., Beanan J.M., Olson R., MacDonald U., Cox A.D., St. Michael F., Vinogradov E.V., Spellberg B., Luke-Marshall N.R., Campagnari A.A. (2013). The K1 Capsular Polysaccharide from *Acinetobacter baumannii* Is a Potential Therapeutic Target via Passive Immunization. Infect. Immun..

[98] De Gregorio E., Del Franco M., Martinucci M., Roscetto E., Zarrilli R., Di Nocera P.P. (2015). Biofilm-associated proteins: News from *Acinetobacter*. BMC Genom..

[99] Skerniškytė J., Karazijaitė E., Deschamps J., Krasauskas R., Armalytė J., Briandet R., Sužiedėlienė E. (2019). Blp1 protein shows virulence-associated features and elicits protective immunity to *Acinetobacter baumannii* infection. BMC Microbiol..

[100] Fattahian Y., Rasooli I., Mousavi Gargari S.L., Rahbar M.R., Darvish Alipour Astaneh S., Amani J. (2011). Protection against *Acinetobacter baumannii* infection via its functional deprivation of biofilm associated protein (Bap). Microb. Pathog..

[101] Rasooli I., Abdolhamidi R., Jahangiri A., Darvish Alipour Astaneh S. (2020). Outer Membrane Protein, Oma87 Prevents *Acinetobacter baumannii* Infection. Int. J. Pept. Res. Ther..

[102] Bahey-El-Din M., Mohamed S.A., Sheweita S.A., Haroun M., Zaghloul T.I. (2020). Recombinant N-terminal outer membrane porin (OprF) of *Pseudomonas aeruginosa* is a promising vaccine candidate against both *P. aeruginosa* and some strains of *Acinetobacter baumannii*. Int. J. Med. Microbiol..

[103] Huang W., Yao Y., Wang S., Xia Y., Yang X., Long Q., Sun W., Liu C., Li Y., Chu X. (2016). Immunization with a 22-kDa outer membrane protein elicits protective immunity to multidrug-resistant *Acinetobacter baumannii*. Sci. Rep..

[104] Luo G., Lin L., Ibrahim A.S., Baquir B., Pantapalangkoor P., Bonomo R.A., Doi Y., Adams M.D., Russo T.A., Spellberg B. (2012). Active and Passive Immunization Protects against Lethal, Extreme Drug Resistant-*Acinetobacter baumannii* Infection. PLoS ONE.

[105] Badmasti F., Ajdary S., Bouzari S., Fooladi A.A.I., Shahcheraghi F., Siadat S.D. (2015). Immunological evaluation of OMV(PagL)+Bap(1-487aa) and AbOmpA(8-346aa)+Bap(1-487aa) as vaccine candidates against *Acinetobacter baumannii* sepsis infection. Mol. Immunol..

[106] Zhang X., Yang T., Cao J., Sun J., Dai W., Zhang L. (2016). Mucosal immunization with purified OmpA elicited protective immunity against infections caused by multidrug-resistant *Acinetobacter baumannii*. Microb. Pathog..

[107] Bolourchi N., Shahcheraghi F., Shirazi A.S., Janani A., Bahrami F., Badmasti F. (2019). Immunogenic reactivity of recombinant PKF and AbOmpA proteins as serum resistance factors against sepsis of *Acinetobacter baumannii*. Microb. Pathog..

[108] Singh R., Garg N., Shukla G., Capalash N., Sharma P. (2016). Immunoprotective Efficacy of *Acinetobacter baumannii* Outer Membrane Protein, FilF, Predicted In silico as a Potential Vaccine Candidate. Front. Microbiol..

[109] Arora S., Thavaselvam D., Kumar A., Prakash A., Barua A., Sathyaseelan K. (2015). Cloning, expression and purification of outer membrane protein (OmpA) of *Burkholderia pseudomallei* and evaluation of its potential for serodiagnosis of melioidosis. Diagn. Microbiol. Infect. Dis..

[110] Weidensdorfer M., Chae J.I., Makobe C., Stahl J., Averhoff B., Müller V., Schürmann C., Brandes R.P., Wilharm G., Ballhorn W. (2016). Analysis of Endothelial Adherence of *Bartonella henselae* and *Acinetobacter baumannii* Using a Dynamic Human Ex Vivo Infection Model. Infect. Immun..

[111] Bentancor L.V., Camacho-Peiro A., Bozkurt-Guzel C., Pier G.B., Maira-Litran T. (2012). Identification of Ata, a Multifunctional Trimeric Autotransporter of *Acinetobacter baumannii*. J. Bacteriol..

[112] Bentancor L.V., Routray A., Bozkurt-Guzel C., Camacho-Peiro A., Pier G.B., Maira-Litrán T. (2012). Evaluation of the Trimeric Autotransporter Ata as a Vaccine Candidate against *Acinetobacter baumannii* Infections. Infect. Immun..

[113] Singh R., Capalash N., Sharma P. (2017). Immunoprotective potential of BamA, the outer membrane protein assembly factor, against MDR *Acinetobacter baumannii*. Sci. Rep..

[114] Garg N., Singh R., Shukla G., Capalash N., Sharma P. (2016). Immunoprotective potential of in silico predicted *Acinetobacter baumannii* outer membrane nuclease, NucAb. Int. J. Med. Microbiol..

[115] Li H., Tan H., Hu Y., Pan P., Su X., Hu C. (2017). Small protein A and phospholipase D immunization serves a protective role in a mouse pneumonia model of *Acinetobacter baumannii* infection. Mol. Med. Rep..

[116] Ronish L.A., Lillehoj E., Fields J.K., Sundberg E.J., Piepenbrink K.H. (2019). The structure of PilA from *Acinetobacter baumannii* AB5075 suggests a mechanism for functional specialization in Acinetobacter type IV pili. J. Biol. Chem..

[117] Ramezanalizadeh F., Owlia P., Rasooli I. (2020). Type I pili, CsuA/B and FimA induce a protective immune response against *Acinetobacter baumannii*. Vaccine.

[118] Qamsari M.M., Rasooli I., Chaudhuri S., Astaneh S.D.A., Schryvers A.B. (2020). Hybrid Antigens Expressing Surface Loops of ZnuD from *Acinetobacter baumannii* Is Capable of Inducing Protection against Infection. Front. Immunol..

[119] Covián C., Fernández-Fierro A., Retamal-Díaz A., Díaz F.E., Vasquez A.E., Lay M.K., Riedel C.A., González P.A., Bueno S.M., Kalergis A.M. (2019). BCG-Induced Cross-Protection and Development of Trained Immunity: Implication for Vaccine Design. Front. Immunol..

[120] Juárez-Rodríguez M.D., Yang J., Kader R., Alamuri P., Curtiss R., Clark-Curtiss J.E. (2012). Live Attenuated Salmonella Vaccines Displaying Regulated Delayed Lysis and Delayed Antigen Synthesis To Confer Protection against *Mycobacterium tuberculosis*. Infect. Immun..

[121] Uppuluri P., Lin L., Alqarihi A., Luo G., Youssef E.G., Alkhazraji S., Yount N.Y., Ibrahim B.A., Bolaris M.A., Edwards J.E. (2018). The Hyr1 protein from the fungus *Candida albicans* is a cross kingdom immunotherapeutic target for *Acinetobacter bacterial* infection. PLoS Pathog..

[122] KuoLee R., Harris G., Yan H., Xu H.H., Conlan W.J., Patel G.B., Chen W. (2015). Intranasal immunization protects against *Acinetobacter baumannii*-associated pneumonia in mice. Vaccine.

[123] Huang W., Wang S., Yao Y., Xia Y., Yang X., Li K., Sun P., Liu C., Sun W., Bai H. (2016). Employing *Escherichia coli*-derived outer membrane vesicles as an antigen delivery platform elicits protective immunity against *Acinetobacter baumannii* infection. Sci. Rep..

[124] García-Quintanilla M., Pulido M.R., Pachón J., McConnell M.J. (2014). Immunization with lipopolysaccharide-deficient whole cells provides protective immunity in an experimental mouse model of *Acinetobacter baumannii* infection. PLoS ONE.

[125] Huang W., Yao Y., Long Q., Yang X., Sun W., Liu C., Jin X., Li Y., Chu X., Chen B. (2014). Immunization against multidrug-resistant *Acinetobacter baumannii* effectively protects mice in both pneumonia and sepsis models. PLoS ONE.

[126] Yang A.-Q., Yang H.-Y., Guo S.-J., Xie Y.-E. (2019). MF59 adjuvant enhances the immunogenicity and protective immunity of the OmpK/Omp22 fusion protein from *Acinetobacter baumannii* through intratracheal inoculation in mice. Scand J. Immunol..

[127] Guo S.J., Ren S., Xie Y.E. (2018). Evaluation of the Protective Efficacy of a Fused OmpK/Omp22 Protein Vaccine Candidate against *Acinetobacter baumannii* Infection in Mice. Biomed. Environ. Sci..

[128] Huang W., Wang S., Yao Y., Xia Y., Yang X., Long Q., Sun W., Liu C., Li Y., Ma Y. (2015). OmpW is a potential target for eliciting protective immunity against *Acinetobacter baumannii* infections. Vaccine.

